# A descriptive study of TB cases finding practices in the three largest public general hospitals in Vietnam

**DOI:** 10.1186/1471-2458-12-808

**Published:** 2012-09-19

**Authors:** Hoa Nguyen Binh, Khanh Pham Huyen, Cornelia Hennig, Hanh Chu Thi, Cuong Le Xuan, Vu Le Thuong, Knut Lönnroth

**Affiliations:** 1 National Tuberculosis Programme Vietnam, Hanoi, Vietnam; 2 World Health Organization (WHO), VietNam Country Office, Hanoi, Vietnam; 3 Bach mai hospital, Hanoi, Vietnam; 4 National Hue hospital, Hue, Vietnam; 5 Cho ray hospital, Ho Chi Minh City, Vietnam; 6 Stop TB Department, WHO, HQ, Geneva, Switzerland

**Keywords:** Vietnam, Tuberculosis, Public-public mix, General hospital, Screening, Diagnosis, Hospital

## Abstract

**Background:**

A project was implemented in 2010 to improve TB notification and TB screening and diagnostic routines in large general hospitals. The aims of present study was to assess baseline TB screening and diagnostic practices in the three largest general hospitals in Vietnam.

**Objectives:**

To assess baseline TB screening and diagnostic practices in the three largest general hospitals in Vietnam.

**Method:**

The study had three elements: 1) Focus group discussions with hospital physicians; 2) review of hospital records and structured interviews of people who had a chest X-ray on any indication; and 3) record reviews and structured interviews of people newly diagnosed with TB.

**Results:**

The most commonly reported diagnostic pathway for pulmonary TB was chest X-ray followed by sputum-smear microscopy. Among 599 individuals who had a chest X-ray performed, 391 (65.1%) had recorded any abnormality, significantly higher in males (73.8%) than in females (54.7%), (p < 0.001), and the proportion was increasing with age (p <0.001). Among those with abnormal chest X-ray, 245 (69.2%) were investigated with sputum smear microscopy, and 49 (20%) were diagnosed with TB, of which 33 (13.5%) were smear-positive.

Of 103 consecutive TB cases enrolled in the study, 92 (90%) had chest X-ray as the initial test. Sixty-three (61.2%) fulfilled the TB suspect criteria based on respiratory symptoms (productive cough >2 weeks).

**Conclusion:**

Chest X-ray is the preferred first test for TB in the largest hospitals in Vietnam. Chest X-ray is a sensitive screening tool for TB, which should be followed by a confirmatory TB test. While the majority of those with chest X-ray abnormalities are investigated with smear-microscopy, the high sputum-smear positivity ratio among them suggests that sputum-smear microscopy is done mainly for persons with quite clear TB signs or symptoms. TB screening and use of confirmatory diagnostic tests on wider indications seem warranted.

## Background

In 2010, the estimated incidence of all forms of tuberculosis (TB) in Vietnam was 199/100,000 population, and the estimated case detection rate of all forms of TB was 54% [1]. The Vietnam National Tuberculosis Control Programme (NTP) is based on the principles of DOTS and the STOP TB strategy, the core control strategy recommended by the World Health Organization (WHO) [2].

In 2006–2007, the Vietnam NTP carried out a combined TB prevalence and tuberculin survey. The survey found a prevalence of smear-positive tuberculosis of 145/ 100,000 (95% CI: 110–180) [3]. This number was 1.6 times higher than the 2006 estimate (89/100,000). Thus, the burden of tuberculosis in Vietnam is considerably greater than previously assumed. A significant number of tuberculosis cases remain undiagnosed and present a potential source of transmission in the community [3].

The prevalence survey showed that about 10% of TB patients were treated in private or public hospitals that do not report to the NTP [4]. This calls for innovative approach to enhance case detection strategies and reporting mechanism in those facilities [5]. A project supported by WHO and funded by the Canadian International Development Agency (CIDA) was implemented in 2010 with the aim to improve case finding by introducing new approaches in hospitals. The project initially targeted the three largest public general hospitals in Vietnam (Bachmai hospital in Hanoi, Choray hospital in Ho Chi Minh City, and Hue National hospital), which together have 5,100 beds and about 7,700 out-patient visits per day. The key element was to ensure full notification and proper treatment and follow up of all TB cases diagnosed in the hospitals. The project also explored ways to improve early TB diagnosis in hospitals by changing TB screening and diagnostic routines, including promotion of sputum smear microscopy (SSM) for all people with possible TB. This paper reports on an assessment of the current procedures for TB screening and diagnosis in the three public general hospitals at a point in time when project implementation had started, but with main focus on full notification of all diagnosed cases. Changes to the screening routines had not yet taken place. Sputum smear microscopy services had been improved and hospital physicians had been encouraged to regularly perform sputum smear microscopy (SSM) for all people with suspected TB, as per NTP guidelines. No other changes had been made to the screening and diagnostic algorithm.

## Methods

### Design

The study had three elements: 1) Focus group discussions with hospital physicians; 2) review of hospital records and structured interviews of people who had a chest X-ray (CXR); and 3) record reviews and structured interviews of people newly diagnosed with TB.

### Sample and data collection

#### Focus group discussions

In each hospital, 10 physicians from the general outpatient and respiratory clinical department participated in focus group discussions, which focused on the common patterns of symptom screening, use of CXR, SSM and other TB tests, and suggestions for improving the TB diagnosis procedure.

#### Review of CXR records and patient interviews

In each hospital, the first 200 adults who had an CXR for any indication from 10^th^ September, 2011, were selected for the study, to assess the proportion that had any abnormality; the proportion that had sputum examinations or other TB diagnostic tests; and the proportion that was diagnosed with TB.

#### Record reviews and interviews of newly diagnosed TB cases

All the adult pulmonary tuberculosis cases diagnosed during two months from the start of the study were interviewed about symptoms as part of the standard clinical assessment at time of diagnosis, and information was collected from records about CXR, SSM and other diagnostic procedures.

### Data analysis

Data were entered in an electronic data file using prewritten entry screens in EpiData Entry (http://www.epidata.dk). The data was exported to Stata 10 for analysis. For categorical variables, χ2 test was used for comparison of the difference in proportions. 95% confidence intervals were used throughout and the level of significance was set at *P* ≤ 0.05.

Adults who had an CXR abnormal were define by radiologist and then verified by clinical doctors. The TB cases were define as those who had at least one sputum smear positive or diagnosed as smear negative or extrapulmonary tuberculosis by clinical doctors in respiratory clinical department in three general hospitals.

### Ethical approval

Risks to study participants were considered minimal, involving only interview time. The diagnosis and treatment was in no way affected by the participation or non-participation in the study. This study required only administrative approval in each hospital. All data was handled with strict confidentially by removing personal information during data entry and storing of original forms in a safe. Verbal consent was obtained from the study subjects.

## Results

**Figure 1:**
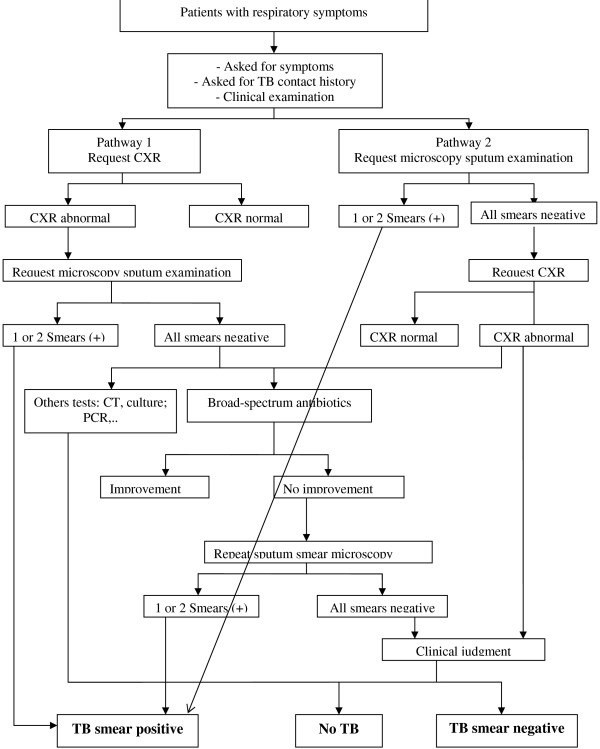
Two tuberculosis diagnostic pathways applied in three largest public general hospitals, Vietnam.

The interviewed physicians reported two pathways to diagnose pulmonary TB in the three hospitals (Figure 1). Pathway 1 starts with CXR, and is followed by SSM if CXR is abnormal and compatible with TB. If SSM is negative, further tests such as chest computed tomography (CT), culture, and/or polymerase chain reaction (PCR) may be done. Pathway 2 starts with SSM, and is followed by CXR if SSM is negative. If CXR shows an abnormality suggestive of TB, physicians make a clinical judgment to diagnose patients as smear-negative TB or no TB (which may be assisted by a diagnostic committee), or prescribe a broad-spectrum antibiotic with subsequent follow-up. In principle, people with productive cough of more than 2 weeks and close TB household contacts should be investigated for TB (in line with the NTP guidelines). However, through pathway 1, people with other signs and symptoms are also investigated with CXR, and therefore CXR abnormality is another, though more implicit criterium for further TB investigations.

In total, 599 individuals who had CXR and 103 adult pulmonary TB patients who diagnosed during two months from start of study were enrolled.

Of 599 individuals who had CXR, 391 (65.1%) had an abnormal CXR result according to the radiologists’ judgment. The proportion of abnormal CXR was significantly higher in males (73.8%) than in females (54.7%), (p < 0.001). This proportion was increasing with age (p < 0.001) (Table 1).

**Table 1:** Proportions of abnormal CXR among patients in three general hospitals, Vietnam

**CXR**		**Total**		**CXR Normal**		**CXR Abnormal**		p value*
		**n**	**%**	**n**	**%**	**n**	**%**	
Total		599	100	208	34.7	391	65.3	
Sex								< 0.001
	Male	332	55.4	87	26.2	245	73.8	
	Female	267	44.6	121	45.3	146	54.7	
Age group								< 0.001
	15-24	54	9	24	44.4	30	55.6	
	25-34	86	14.4	44	51.2	42	48.8	
	35-44	82	13.7	25	30.5	57	69.5	
	45-54	120	20	46	38.3	74	61.7	
	55-64	121	20.2	34	28.1	87	71.9	
	65+	136	22.7	35	25.7	101	74.3	
Age (years) mean+/− sd		50.1 (17.5)		46.5 (17.3)		52.0 (17.4)		< 0.001
Hospital								< 0.001
	Bachmai	198	33.1	56	28.3	142	71.7	
	Hue	199	33.2	32	16.1	167	83.9	
	Choray	202	33.7	120	59.4	82	40.6	

CXR = Chest X-ray.

* P value by Chi square test.

Of 391 patients with an abnormal CXR, 354 were interviewed. Of those, 245 (69.2%) had sputum smear examination, Among them, 33 (13.5%) had at least one positive smear and a total of 49 (20%) were diagnosed with TB (Table 2). The proportion of smear positive among sputum examination was 27.3% in Hue, 4.5% in Bachmai hospital and 2.8 in Choray hospital.

**Table 2:** Proportion of sputum examination and TB diagnosis among abnormal CXR in three general hospitals, Vietnam

**Hospitals**	**Abnormal CXR interviewed**	**Request sputum examination**		**TB diagnosis**		**Smear negative**	**Smear positive**
	**n**	**n**	**%**	**n**	**%**	**n**	**n**
Total	354	245	69.2	49	20.0	16	33
Bach mai	134	110	82.1	15	13.6	10	5
Hue	166	99	59.6	33	33.3	6	27
Cho ray	54	36	66.7	1	2.8	0	1

(n = 354).

CXR = Chest X-ray; TB = tuberculosis.

A total of 103 adult pulmonary TB patients were enrolled in the study (including the 48 TB cases diagnosed from the CXR cohort). These were 69 (67%) smear-positive and 34 (33%) smear-negative pulmonary TB cases. Sixty-three (61.2%) met the criteria of a TB suspect of Vietnam NTP (cough with sputum for more than 2 weeks). One hundred and two (99%) of the TB patients had a CXR examination, and for 92 (90%) CXR was the first investigation. SSM was performed on 96 (93%) of the TB patients (Table 3).

**Table 3:** Proportions of CXR, sputum examination tests in adult pulmonary TB patients in three general hospitals, Vietnam

		**Total**		**Bachmai**		**Hue**		**Choray**		**P value**
		**N**	**%**	**N**	**%**	**N**	**%**	**N**	**%**	
Total		103		17		58		28		
Productive cough										< 0.001
	Yes	63	61.2	7	41.2	49	84.5	7	25.0	
	No	40	38.8	10	58.8	9	15.5	21	75.0	
Diagnostic procedure										0.257
	CXR before sputum examination	92	90.2	14	82.4	55	96.5	23	82.1	
	Sputum examination before CXR	3	2.9	0	0	1	1.8	2	7.1	
	No information	7	6.9	3	17.6	1	1.8	3	10.7	
CXR during diagnosis										
	Yes	102	99.0	17	100.0	58	100.0	27	96.4	0.259
	No information	1	1.0	0	0.0	0	0.0	1	3.6	
Sputum examination during diagnosis										0.042
	Yes	96	93.2	16	94.1	52	89.7	28	100.0	
	No	6	5.8	0	0.0	6	10.3	0	0.0	
	No information	1	1.0	1	5.9	0	0.0	0	0.0	

CXR = Chest X-ray;* P value by Chi square test.

## Discussion

The record review and patient interviews confirmed that CXR is the preferred first investigation for TB in the three hospitals. This does not necessarily reflect a systematic approach to TB screening and diagnosis, but probably mirrors the fact that CXR is a common general screening and diagnostic tool for respiratory illnesses, which is widely available and used in general hospitals. About two thirds of all patients with abnormal CXR were further investigated for TB with SSM.

Among persons who had a CXR examination, the proportion with any abnormality was 65%. The proportion was highest among those over the age of 65 years and among men, consistent with findings of the first national TB prevalence survey in Vietnam [3].

Using any CXR abnormality as screening criterium for further TB diagnosis in these hospitals implies TB testing of the majority of those who have a diagnostic CXR on any indication, and thus TB testing of a large proportion of all patients in respiratory and other out-patient departments (OPDs). On the one hand, this can be expensive and overburden laboratories. However, the TB yield among these patients is high, even among people who do not have typical TB symptoms, as demonstrated in the national TB prevalence survey in Vietnam and elsewhere [3,6].

In this study, the proportion of TB cases among people with abnormal CXR was 20% and the sputum-smear positivity ratio was 13.5%. This is double the culture positivity ratio among people with abnormal CXR in the TB prevalence survey in Vietnam (6.6%) [3]. There were large differences in the positivity of abnormal CXR who had sputum examination in the three hospitals, 27.3% in Hue, 4.5% in Bachmai hospital and 2.8 in Choray hospital. The reasons for higher proportions of smear positive among abnormal CXR who had sputum examination in Hue than in Bachmai hospitals and Choray hospital may be explained by the fact that Hanoi and HCMC have TB hospitals (National Lung hospital and Hanoi TB and Lung disease hospital in Hanoi, and Pham Ngoc Thach hospital in HCMC), while Hue has no provincial TB hospital, only a provincial social disease and prevention center, so many TB suspects go to general Hue hospital for examination. Another reason for the difference in positivity may be that the study sample came from the general OPD in Choray while the study sample came from the chest OPD in Hue and Bachmai.

The finding of more than 30% of people with abnormal CXR not being investigated with SSM, in combination with a high proportion of TB among those investigated suggests that there are still missed opportunities to improve early TB detection in the hospital sector in Vietnam. Aiming for SSM in close to 100% of those with abnormal CXR findings would likely improve early TB detection. Using a more sensitive test than SSM, such as GeneXpert MTB/Rif would improve case detection further, while also reducing the number of smear-negative patients going through a complicated diagnostic work-up, which has low diagnostic precision, especially if the final diagnosis is based on CXR finding and clinical picture only [7,8].

Is it rational to do CXR before a bacteriological TB test in hospitals? There are a couple of arguments in favor: First, CXR is a good tool for ruling out pulmonary TB. The high sensitivity of CXR to detect culture positive TB has been demonstrated also in Vietnam [3]. Second, TB symptoms are not specific [9,10]. Therefore many possible differential diagnosis need to be considered, and CXR is a screening and diagnostic tool for several respiratory conditions. Third: final CXR results can normally be made available more quickly than results of 2–3 SSM examinations, minimizing the risk of delay and defaulting during the diagnostic process. Fourth, CXR screening can minimize the burden on laboratories. A large number of people come to hospital OPDs, and a high proportion of them have cough. If these hospitals were to strictly follow the diagnostic algorithm recommended by the Vietnam NTP, a large number of SSM would be required, and then CXR examination for those with negative SSM, which would be the vast majority. This would lead to a large burden for the laboratory of these hospitals and not much less burden for the radiology department. However, a similar argument could be made about the burden on the radiology department when CXR is the primary test. Furthermore, if a very high proportion of patients have an abnormal CXR, most patients would anyway need to have both CXR and SSM.

Arguments against using CXR as a screening tool include higher cost than SSM (at least for conventional X-ray), and lack of standards and quality control of x-ray machines, films and reading [11]. In addition, in the absence of a highly sensitive and specific confirmatory TB test, it can generate false positive smear-negative TB cases, even if backed up by good diagnostic practices [12]. However, the CXR pros-and-cons equation changes with the potential for introducing GeneXpert MTB/Rif, a more sensitive test than SSM, more specific than CXR, but much more expensive than both [7,8,13,14]. Pre-screening with CXR to minimize use of Xpert MTB/Rif would save more costs than pre-screening before SSM. Also, the risk of generating many false positive smear-negative TB cases through initial CXR screening would diminish with the use of XpertMTB/Rif due to the capacity of the test to both rule in and rule out TB. There are already diagnostic committees in place in the three hospitals, which have probably contributed to the low proportion of smear-negative TB cases found in the present study. However, diagnosing smear-negative TB based on CXR and clinical picture is always imprecise. Our study has some limitations. Firstly, the study was designed to describe current practices, and not to assess accuracy, sensitivity and specificity of the different diagnostic pathways. The study did not include a validation of diagnosis. Therefore, the precision of the procedures and the proportion of false negative and false positive cases are unknown. This study did not assess treatment uptake and outcomes among TB patients diagnosed in hospitals. This will be part of the subsequent project evaluation.

## Conclusions

The most common pathway of diagnosing pulmonary TB in the three largest general hospitals in Vietnam is to first do a CXR, and then SSM. This seems a feasible and suitable practice and should be considered in all general hospitals in Vietnam. CXR is a sensitive TB screening tool and is valuable also for screening and diagnosis of other respiratory conditions. It therefore makes sense to use CXR as a general screening tool in a chest OPD/ward. Since this is common practice already, there is an opportunity to intensify TB diagnosis among all people screened positive for any CXR abnormality. With the demonstrated high proportion of TB patients among people with any CXR abnormality, all persons with abnormal CXR should be investigated by SSM, or other bacteriological TB tests, at least in settings with high TB prevalence. With the introduction of GeneXpert MTB/Rif, pre-screening with CXR can save costs, while the confirmation of TB with GeneXpert MTB/Rif after CXR can help minimize the risk of CXR for screening or diagnosis contributing to diagnosis of false-positive TB cases.

## Competing interests

The author(s) declare that they have no competing interests.

## Authors’ contributions

All the authors contributed to the design of the study. NBH, PHK, CH, CTH, LXC, LTV were responsible for implementing the study, data collection and data entry. PHK, CH and KL were responsible for the conception and overall supervision of the quality of data collection, data entry and analysis. NBH was responsible for data analysis. NBH and KL wrote the first draft of the paper and all co-authors contributed to the writing of the final paper. All authors read and approved the final manuscript. KL is guarantor of the paper.

## Pre-publication history

The pre-publication history for this paper can be accessed here:

http://www.biomedcentral.com/1471-2458/12/808/prepub
